# Conversion of Minimally Invasive Liver Resection for HCC in Advanced Cirrhosis: Clinical Impact and Role of Difficulty Scoring Systems

**DOI:** 10.3390/cancers15051432

**Published:** 2023-02-23

**Authors:** Federica Cipriani, Francesca Ratti, Gianluca Fornoni, Rebecca Marino, Antonella Tudisco, Marco Catena, Luca Aldrighetti

**Affiliations:** 1 Hepatobiliary Surgery Division, IRCCS San Raffaele Scientific Institute, 20132 Milan, Italy; 2 Faculty of Medicine and Surgery, Vita-Salute San Raffaele University, 20132 Milan, Italy

**Keywords:** laparoscopic liver resection, minimally invasive liver resection, conversion, cirrhosis, Child B, portal hypertension, difficulty score

## Abstract

**Simple Summary:**

It is essential to consider the specific impact that conversion can have in a context where MILR is so positively determinant, that is, hepatocellular carcinoma. It has not yet been specifically investigated what impact conversion may have in case of advanced cirrhosis, which is the central risk factor for specific postoperative complications and the context in which the loss of minimally invasive benefits can be particularly harmful. This study showed that conversion in the setting of advanced cirrhosis can be associated with non-inferior outcomes compared to compensated cirrhosis, provided careful patient selection is applied. Difficulty scoring systems may help in identifying the most appropriate candidates to maintain satisfactory outcomes, even in case of conversion, and become helpful in multidisciplinary treatment decisions.

**Abstract:**

Background: Minimally invasive liver resections (MILRs) in cirrhosis are at risk of conversion since cirrhosis and complexity, which can be estimated by scoring systems, are both independent factors for. We aimed to investigate the consequence of conversion of MILR for hepatocellular carcinoma in advanced cirrhosis. Methods: After retrospective review, MILRs for HCC were divided into preserved liver function (Cohort-A) and advanced cirrhosis cohorts (Cohort-B). Completed and converted MILRs were compared (Compl-A vs. Conv-A and Compl-B vs. Conv-B); then, converted patients were compared (Conv-A vs. Conv-B) as whole cohorts and after stratification for MILR difficulty using Iwate criteria. Results: 637 MILRs were studied (474 Cohort-A, 163 Cohort-B). Conv-A MILRs had worse outcomes than Compl-A: more blood loss; higher incidence of transfusions, morbidity, grade 2 complications, ascites, liver failure and longer hospitalization. Conv-B MILRs exhibited the same worse perioperative outcomes than Compl-B and also higher incidence of grade 1 complications. Conv-A and Conv-B outcomes of low difficulty MILRs resulted in similar perioperative outcomes, whereas the comparison of more difficult converted MILRs (intermediate/advanced/expert) resulted in several worse perioperative outcomes for patients with advanced cirrhosis. However, Conv-A and Conv-B outcomes were not significantly different in the whole cohort where “advanced/expert” MILRs were 33.1% and 5.5% in Cohort A and B. Conclusions: Conversion in the setting of advanced cirrhosis can be associated with non-inferior outcomes compared to compensated cirrhosis, provided careful patient selection is applied (patients elected to low difficulty MILRs). Difficulty scoring systems may help in identifying the most appropriate candidates.

## 1. Introduction

Minimally invasive liver resections (MILRs) have seen a considerable diffusion as an alternative to the traditional open approach thanks to the evidence of positive effects, currently well known, for the postoperative course [1,2,3,4,5]. Since its onset, MILRs have been shown to be particularly beneficial for patients affected by hepatocellular carcinoma (HCC), being specifically associated with reduced postoperative ascites and hepatic insufficiency [6,7,8,9,10,11,12,13]. 

When approaching liver resection with minimally invasive techniques, it is known that conversion to laparotomy may be necessary to complete the operation safely [14,15,16]. Although this event should not necessarily be considered a complication or failure, it is well known that conversion actually has a non-negligible impact on results, being associated with inferior perioperative outcomes compared to both successfully completed MILRs and upfront open resections [17,18,19,20,21]. Consequently, it is recognized—and should be expected—that converted resections may at least lose some of the benefits of adopting a minimally invasive approach [22,23,24,25]. Therefore, it is essential to take into account the specific impact that conversion can have in a context where MILR is so positively determinant, that is, HCC. 

It should also be considered that MILRs in cirrhosis are among the most complex minimally invasive liver surgeries and simultaneously exposed to a significant possibility of conversion given the reported role of cirrhosis as an independent factor for both conversion and MILR difficulty [26,27]. Furthermore, the MILR complexity itself should be considered a predisposing factor for conversion, which can be reliably estimated by existing complexity scoring systems [26,28]. 

Given the current trends to increasingly implement MILRs in cirrhosis and to consider minimally invasiveness as a potential means for an extension of HCC resective indications, the aim of this study was to investigate the consequence of conversion in the specific setting of advanced cirrhosis. In fact, it has not yet been investigated what impact conversion may have in the case of advanced cirrhosis, which represents the central risk factor for specific postoperative complications and the context in which the loss of minimally invasive benefits can be particularly harmful. The hypothesis was that advanced cirrhosis could adversely affect perioperative outcomes in case of conversion, with even inferior results compared to patients with compensated chronic liver disease. The ultimate purpose is to add useful knowledge helpful in the refinement of indications to resection for HCC and in line with technical and technological development, which is presently an ongoing process.

## 2. Materials and Methods

### 2.1. Study Design

Data of consecutive patients undergoing MILR at a single hepatobiliary center (January 2005–August 2022) were retrospectively reviewed for the purpose of this case series study. Patients affected by histologically proven HCC undergoing MILR were selected. 

**Figure 1:**
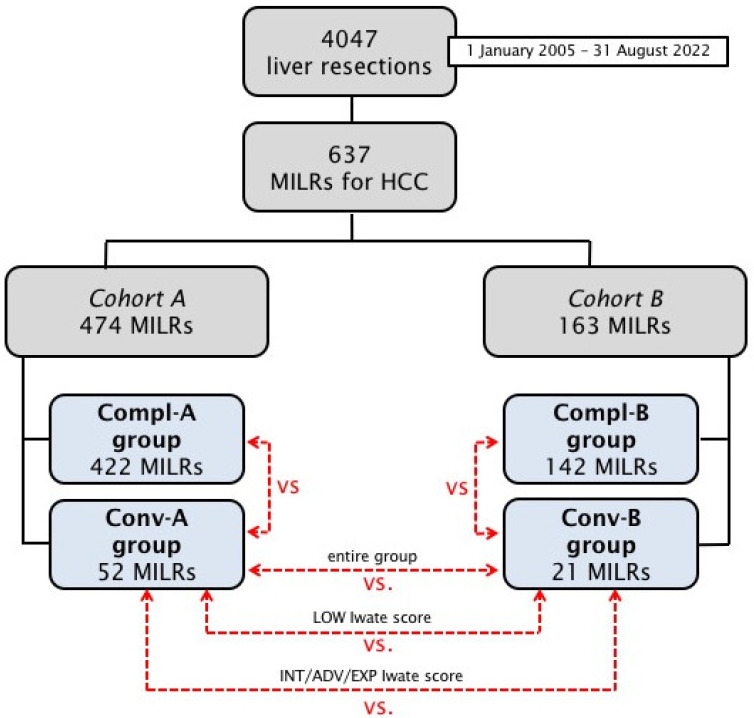
The study design. HCC stands for hepatocellular carcinoma. MILR stands for minimally invasive liver resection.

The study design is depicted in Figure 1. 

Patients were separated into two cohorts according to the severity of chronic liver disease: cohort A including patients with preserved liver function (i.e., Child A without portal hypertension) and cohort B including patients with advanced liver disease (i.e., Child B/C cirrhosis or any Child stage associated with clinically significant portal hypertension defined as gastroesophageal varices on endoscopy or platelet count < 100 × 10^9^/L in the presence of splenomegaly > 120 mm) [29,30]. 

Each cohort was further classified into a “minimally invasive completed” or “converted” group based on the occurrence of conversion to open during MILR. The two groups of converted patients (named Conv-A and Conv-B) were compared with minimally invasive completed patients (named Compl-A and Compl-B groups). To further enhance the analysis, liver resections were stratified in classes of increasing complexity using the Iwate criteria, a difficulty multiparametric scoring system specifically produced for MILR and already validated as the most reliable tool among difficulty scoring systems to predict conversion risk for HCC. Indeed, Lin and colleagues recently reported that, among existing difficulty scoring systems for MILR, only the Iwate criteria were able to predict conversion to laparotomy in the specific setting of HCC [26,28]. 

The analysis followed a three-step process:

1. Minimally invasive completed and converted patients in each cohort were compared (Compl-A vs. Conv-A and Compl-B vs. Conv-B) so as to test in our series the loss of advantage of conversion for any severity of chronic liver disease separately.
2. Converted patients of each cohort were compared (Conv-A vs. Conv-B) to test for differences in outcomes for converted patients with their severity of chronic liver disease.
3. Converted patients of each cohort were compared (Conv-A vs. Conv-B) selectively for low Iwate difficulty level and intermediate/expert/advanced Iwate difficulty level to test for differences in outcomes for converted patients with their severity of chronic liver disease in different settings of MILR complexity.

### 2.2. Outcome Measures

Data regarding the characteristics of patients, disease and perioperative course were collected. The analyzed baseline features included demographics, MELD score, ASA score, Charlson Comorbidity Index, background liver status, portal hypertension, baseline liver function, number of lesions, size of largest lesion (cm) and extent of resection. 

Perioperative parameters were registered: operative time (minutes), estimated blood loss (mL), red blood cells and fresh frozen plasma transfusions, Pringle maneuver, completeness of resection (R0), conversion to open and reasons, mortality and both general and liver-specific morbidity, post-discharge readmission and length of stay (days). 

### 2.3. Indications, Surgical Technique and Perioperative Management

The standard assessment of HCC patients included clinical examination and laboratory (liver function, serum tumor markers), endoscopic (esophagogastroduodenoscopy) and radiological tests (abdominal ultrasonography, thoracoabdominal contrast enhanced imaging) to assess liver function according to Child–Pugh score, signs of portal hypertension and tumor characteristics and staging. For all patients deemed eligible to liver resection, the treatment strategy was systematically evaluated at weekly multidisciplinary hepatobiliary meetings (inclusive of hepatobiliary surgeon, hepatologist and medical oncologist opinions) in order to validate the indication to surgery and technique. 

Pure laparoscopic or robotic procedures were attempted in all patients, and no hybrid techniques were used. Our technique for MILR has been previously described [1,31,32,33,34]. Conversions were all performed directly to laparotomy, i.e., the standard, so as to protect the patient from complications related to late/emergency conversions [35]. 

Patients were managed with a perioperative fast-track protocol to enhance functional recovery. Functional recovery was considered achieved when all following criteria were met: adequate pain control with oral analgesics; independently mobile; tolerance of solid food; normal or improving blood tests; and no intravenous fluids. Patients are considered for discharge when both the functional recovery and patient’s agreement are obtained. A specific analgesic and store red blood cells protocol is followed for pain management [36,37,38]. 

### 2.4. Definitions

Type of liver resections were classified according to the Brisbane 2000 Terminology of Liver Anatomy and Resections [39]. Postoperative morbidity, mortality and readmission were reviewed at 90 days after surgery and complications were graded according to the Clavien–Dindo classification of postoperative complications [40]. Liver-specific complications were liver failure (liver failure as an increased international normalized ratio and concomitant hyperbilirubinemia on or after postoperative day 5 [41]); ascitic decompensation (abdominal drainage above 10 mL/kg body weight/day after postoperative day 3 [42]); biliary leakage (bilirubin concentration in the drainage above three-fold of serum total bilirubin on or after pod 3) or the need for radiologic or operative intervention from a biliary collection or bile peritonitis [43].

### 2.5. Statistics

Data were expressed as median (with interquartile ranges) for continuous variables as their distribution was skewed (Shapiro-Wilk Test); categorical variables were expressed as absolute values and proportions. Continuous variables were compared using the nonparametric Mann–Whitney test; categorical variables were compared through the Fisher’s exact or chi-square test. Statistical significance was set at *p* < 0.05. All analyses were performed using the statistical package SPSS 22.0 (SPSS, Chicago, IL, USA). 

The work has been approved by the institutional Ethical Committee.

## 3. Results

The study population consisted of 637 MILRs for HCC (selected from a global pool of 4047 liver resections), divided into 474 pertaining to Cohort A and 163 to Cohort B (Figure 1). 

The baseline characteristics of patients and operations included in Cohort A and B are depicted in Table 1. 

There were no statistically significant differences in terms of age, gender, ASA score, Charlson Comorbidity Index, etiology of liver disease, MELD score, number of tumors and history of previous liver resection. Groups were well balanced between the approaches in terms of comorbidities, features of the liver parenchyma, etiology of liver disease and previous liver resection. Between Cohort A and B, the following parameters recorded a statistically significant difference: proportion of histological type of chronic liver disease, tumor locations, presence of varices, ascites or thrombocytopenia, tumor size, operation type and Iwate difficulty level. In particular, the proportion of MILRs classified as of “advanced/expert” difficulty was 33.1% in Cohort A and 5.5% in Cohort B (*p* = 0.025). Notably, there was no statistically significant difference in the median MELD score for both groups despite being higher in Cohort B; moreover, both groups were within 9 points, which is a value associated with acceptable risk liver surgery in terms of perioperative morbidity and mortality [44]. 

Cohort A resections were further classified into 422 Compl-A and 52 Conv-A (10.9% conversion rate) and Cohort B resections into 142 Compl-B and 21 Conv-B (12.9% conversion rate) (Figure 1). The cumulative conversion rate resulted in 11.4% (n = 73), and reasons of conversion had a comparable incidence among the two cohorts: the most frequent reason in both was bleeding or unsatisfactory hemostasis (40.4% and 52.4% of conversions, *p* = 0.475) followed by any concern of oncologic inadequacy (compromised margins or any doubt on radical resection), difficult adhesiolysis, unsatisfactory biliostasis and anesthesiological problems (Table 2).

### 3.1. MILR in Patients with Preserved Liver Function: Completed versus Converted

Conv-A showed higher amount of blood loss (400 vs. 100 mL, *p* = 0.009) and incidence of fresh frozen plasma transfusions (21.1% vs. 3.3%, *p* = 0.004) than Compl-A. Moreover, global morbidity (23.1% vs. 11.8%, *p* = 0.018), grade 2 complications (13.4% vs. 6.4%, *p* = 0.016), ascites (17.3% vs. 5.0%, *p* = 0.004), postoperative liver failure (9.6% vs. 2.4%, *p* = 0.018) and pleural effusion (7.7% vs. 2.8%, *p* = 0.015) were more frequent in Conv-A group, as well as length of stay being longer (7 vs. 5 days, *p* = 0.007) and the readmission rate higher (7.7% vs. 1.9%, *p* = 0.036).

The rest of the parameters demonstrated nonsignificant differences (Table 3).

### 3.2. MILR in Patients with Advanced Chronic Liver Disease: Completed versus Converted

As with cohort B, the parameters associated with a statistically significant difference in cohort A showed less favorable results for converted than completed MILRs: amount of blood loss (550 vs. 250 mL, *p* = 0.007), fresh frozen plasma transfusions (28.6% vs. 6.3%, *p* = 0.003), global morbidity (28.6% vs. 12.7%, *p* = 0.002), grade 2 complications (14.3% vs. 7.0%, *p* = 0.021), ascites (19.0% vs. 4.9%, *p* = 0.008), postoperative liver failure (9.5% vs. 2.8%, *p* = 0.034), pleural effusion (9.5% vs. 4.2%, *p* = 0.039), length of stay (8 vs. 5 days, *p* = 0.005) and readmission rate (9.5% vs. 3.5%, *p* = 0.024). In addition, Conv-B also showed a higher incidence of grade 1 complications (14.3% vs. 4.2%, *p* = 0.032).

The rest of the parameters demonstrated nonsignificant differences (Table 4).

### 3.3. Converted MILR: Patients with Preserved Liver Function versus Patients with Advanced Chronic Liver Disease (Whole Cohorts) 

Conv-A and Conv-B groups showed similar perioperative outcomes as a statistically significant difference was recorded only for Grade 1 complications, which were higher for Conv-B patients (14.3% vs. 3.8%, *p* = 0.030). Indeed, all the other parameters showed comparable results between the two groups (Table 5).

### 3.4. Converted MILR: Patients with Preserved Liver Function versus Patients with Advanced Chronic Liver Disease (Low Iwate Difficulty Level)

Conv-A and Conv-B groups showed similar perioperative outcomes as a statistically significant difference was recorded only for Grade 1 complications, which were higher for Conv-B patients (12.5% vs. 6.6 %, *p* = 0.024). Indeed, all the other parameters showed comparable results between the two groups (Table 6).

### 3.5. Converted MILR: Patients with Preserved Liver Function versus Patients with Advanced Chronic Liver Disease (Intermediate/Expert/Advanced Iwate Difficulty Level)

Conv-B showed a higher amount of blood loss (700 vs. 400 mL, *p* = 0.029), incidence of red blood cell transfusions (7.7% vs. 5.4%, *p* = 0.034) and fresh frozen plasma transfusions (38.5% vs. 24.3%, *p* = 0.007) than Conv-A. Moreover, global morbidity (38.5% vs. 24.3%, *p* = 0.038), grade 2 complications (23.1% vs. 13.5%, *p* = 0.031), ascites (30.8% vs. 21.6%, *p* = 0.025), collection (7.7% vs. 2.7%, *p* = 0.034) and pleural effusion (15.4% vs. 10.8%, *p* = 0.037) were more frequent in Conv-B group, as well as length of stay being longer (9 vs. 6 days, *p* = 0.022).

The rest of the parameters demonstrated nonsignificant differences (Table 7).

## 4. Discussion

MILRs in cirrhosis are universally recognized as challenging. It must be considered that several risk factors for the conversion and difficulty of MILRs have been investigated and various studies have reported the independent role of cirrhosis in both contexts [26,27]. Therefore, MILRs in cirrhosis are regarded as among the most complex minimally invasive liver operations and simultaneously exposed to a relevant possibility of conversion. The need to analyze MILRs is based on the fact that current trends are to increasingly implement MILRs worldwide. This is due to its favorable effects of reduced blood loss, morbidity, hospitalization and favoured pain control, together with less postoperative inflammatory response. There is no evidence that MILRs can prevent HCC development and an oncological long-term advantage cannot be accounted among the demonstrated benefits. However, MILR for HCC is specifically associated with important short-term benefits which are the reduced incidence of postoperative ascites and hepatic insufficiency. These advantages have led HCC to constitute the prevalent indication for MILR [10,11,12,13]. Indeed, despite the disadvantages of MILR, which are technical hurdles for the surgeon linked to the loss of direct manual action as challenging bleeding control and intraoperative staging, many studies have shown adequate and favorable results provided the learning curve is completed. The status of current widely used techniques other than MILR include open liver resection, liver transplantation and ablation as options for HCC according to tumor- and liver-related factors. Minimally invasive approaches have entered this scenario not only as an alternative technique to perform curative treatments but also as a potential means of extending the indications to HCC resection. With these premises, this study specifically investigated the outcomes associated with MILR conversion in patients with HCC and liver cirrhosis, with the purpose of specifically investigating outcomes in the setting of advanced cirrhosis given that conversion is not avoidable in a certain proportion of MILRs and considering its potentially harmful effect. The novelty is that this knowledge would help in the mindful process of the refinement of outcomes and indications according to technical and technological development. 

Compared to successfully completed MILRs, conversion is known to be associated with worse perioperative outcomes and also inferior overall survival in case of malignant diagnosis [17,18,19,20,23,24]. To our knowledge, three previous studies published between 2019 and 2021 investigated the impact of conversion in the specific oncological context of HCC. Stiles and colleagues [23] identified nearly 1000 patients undergoing attempted MILR within a national American cancer database, whereas Lee and Shin and their colleagues [24,25] analyzed nearly 300 MILRs performed at two separate Korean institutions. In all three studies, successfully completed MILRs were compared with patients converted. Despite comparable mortality rates, these were associated with poorer perioperative outcomes including longer postoperative hospitalization, higher blood loss and transfusion rates, longer operative times and higher readmission and morbidity rates including ascites. Although it is clear from these data that converted MILRs for HCC may lose some of the benefits of the minimally invasive approach such as in other disease contexts, the issue of the impact of conversion in relationship with the severity of cirrhosis remained unexplored. Thus, the question persisted unanswered whether MILR conversion in advanced cirrhosis may be held to even inferior outcomes than in the setting of compensated liver disease. 

By separately comparing successfully completed with converted MILRs for compensated and advanced cirrhosis, this study confirmed that conversion has inferior results for both compensated and advanced cirrhotic patients. In both cases, converted patients exhibited greater blood loss, higher transfusion rates, longer hospitalization and higher morbidity and readmission rates. Notably, the incidence of pleural effusion, ascites and postoperative liver failure was higher, which are major concerns for patients affected by chronic liver disease given the potential negative impact of short-term survival. Always, when allocating a patient to liver resection in consideration of the possible beneficial course linked to a minimally invasive approach, the possibility of conversion, which is expected to provide a postoperative course similar to that of open resection, has to be considered. This is of utmost importance when the feasibility of a minimally invasive approach weighs significantly in favor of liver resection as the therapeutic choice. Progressive literature has demonstrated the significant advantages of the minimally invasive approach for HCC surgery, precisely in terms of the reduction of ascites and postoperative liver failure, i.e., the elements that traditionally most limit the choice of resection as the treatment option. Moreover, during the years, this advantage has also resulted in patients with advanced cirrhosis, leading them to have a postoperative course similar to that of compensated patients [8,12,45]. This evidence has led to hypothesize and also consider a formal expansion of resective indications for more fragile patients [13]. However, a tendency of this type cannot disregard the consideration that the possibility of conversion exists and cannot be canceled, nor can its possible effects. Therefore, the choice of allocating a patient to resection by virtue of the feasibility of a minimally invasive operation must be a criterion proposed with an awareness of the limits and applied to selected patients if this is based on an extension of the indications.

This study took the available evidence a step further: it showed that conversion in patients with advanced cirrhosis can have similar—and not inferior—results to patients with compensated cirrhosis, provided adequate patient selection is applied. In fact, the comparative analysis between the two groups of converted patients (entire cohort) resulted in non-significant statistical differences regarding perioperative outcomes (the only exception was the incidence of grade 1 complications, which was higher in patients with advanced cirrhosis). Although well accepted, this finding was not entirely expected on the assumption that a laparotomy in the patient with decompensated cirrhosis or with portal hypertension is generally associated with less brilliant results than in the patient with compensated cirrhosis, in particular with a higher rate of postoperative complications and a prolonged stay. Therefore, in interpreting the result of this analysis, the difficulty profile of the resections performed has a logical explanatory role. It is immediate to note that only an extremely limited portion of the cohort of patients with advanced cirrhosis underwent complex resections (understood as advanced or expert level) with a clear prevalence of resections of low and intermediate difficulty; at the same time, the complexity of the resections was much more homogeneous in the cohort of patients with compensated cirrhosis, of which more than 30% received a complex resection. A more contained difficulty profile of resections appears able to counteract the clinical effect that conversion can have in patients with advanced cirrhosis, keeping the average impact similar to that of compensated patients undergoing a wider and less restrictive range of procedures. This is further supported by the results of the analysis comparing conversion in patients with compensated and advanced cirrhosis when resections are stratified for difficulty. Indeed, the comparative analysis between the two groups of converted low difficulty MILRs resulted in non-significant statistical differences regarding perioperative outcomes (the only exception was again the incidence of grade 1 complications, which was higher in patients with advanced cirrhosis). Instead, the comparison of converted more difficult MILRs (intermediate/advanced/expert) resulted in several worse perioperative outcomes for patients with advanced cirrhosis such as higher blood loss, transfusion rates, global morbidity and grade 2 complications rates including ascites, collection and pleural effusion, as well as a longer length of stay. As such, it is clear that advanced cirrhotics undergoing intermediate/advanced/expert MILR is a category of patients that pays a significant price of conversion and that the difficulty of MILR is a factor that should be taken into significant account in the process of patient selection. It follows that the difficulty scoring system can be a very useful tool in the decision-making of proposing minimally invasive surgery to patients with advanced cirrhosis: it may help identify those patients for whom broadening the indications allows maintaining satisfactory outcomes, including the potential conversion effect (patients elected to low difficulty MILR), and may be at the basis of reasoned and aware modern resective indications. The significance of these novel results support the idea that the process of proposing expanded resective indications for HCC in view of the feasibility of minimally invasive operations must include criteria proposed with an awareness of the limitations and applied to selected patients, and a difficulty scoring system for MILR can play a useful role for patient selection.

Regarding the incidence of MILR conversions in HCC, the rates reported in the literature vary greatly as for MILR in general. Stiles, Shin and Lee reported rates of 18.0%, 6% and 4%, respectively, and our study’s rate falls somewhere between these values at 11%. We found the conversion rate was not different between the two groups as well as the incidence of bleeding as a cause, despite—as in the other studies—being the most frequent reason for conversion. This is obviously related to the aptitude of cirrhotic parenchyma to bleed more than healthy liver and to the known frequent coagulation disorders observed in cirrhotic patients. [24,25]. It has been also reported that patients who experience emergent conversion due to an intraoperative complication suffered even worse perioperative outcomes than those undergoing elective conversion [21]. The limited number of conversions, due to the single-center design of this study, precluded this specific analysis which can be the subject of a multi-center analysis together with the reasons of conversion. We cannot exclude that larger sample sizes could find higher rates of conversions in the advanced cirrhotic group, especially due to difficult hemostasis or bleeding, highlighting the role of cirrhosis status rather than the presence of cirrhosis itself in this setting. The other limitation to be acknowledged for this study is the retrospective design, which carries in itself the burden of selection bias and a possible influence on some results. However, it is clear that selection bias is itself a premise for satisfactory outcomes in these particular categories of patients. 

This study sets the stage for a systematization of patient selection, and we believe that future studies should continue in this direction in order to achieve a thoughtful and accurate expansion of the indications, ideally validated within guidelines.

## 5. Conclusions

In conclusion, conversion during MILR for HCC represents a loss of advantage with respect to successfully completed MILRs, and the risk and impact of conversion should be accurately estimated when proposing to expand the indications to liver resection for HCC in view of a minimally invasive approach. Conversion in the setting of advanced cirrhosis can be associated with non-inferior outcomes compared to compensated cirrhosis, provided careful patient selection is applied (patients elected to low difficulty MILR). Difficulty scoring systems may help identify the most appropriate candidates to maintain satisfactory outcomes, even in case of conversion, and become useful in multidisciplinary treatment decisions.

## Figures and Tables

**Table 1:** Baselines of MILRs for HCC in Child A and in advanced cirrhosis (Child B and Child A/B with portal hypertension) patients.

	Cohort A n = 474	Cohort B n = 163	*p* Value
Age, years	71 ± 5	73 ± 6	0.612
Gender [M/F], n (%)	208/266 (43.9/56.1%)	83/80 (50.9/49.1%)	0.845
MELD score, points	7	8	0.324
ASA score [1–2/3–4], n (%)	279/195 (58.8/41.2%)	79/84 (48.5/51.5%)	0.292
Charlson Comorbidity Index, points	9	12	0.478
Etiology of chronic liver disease, n (%) Viral Alcoholic Metabolic Other/unknown	73 (15.4%) 106 (22.4%) 183 (38.6%) 112 (23.6%)	30 (18.4%) 36 (22.1%) 41 (25.1%) 56 (34.3%)	0.658
Liver parenchyma, n (%) Mild fibrosis (F0-1) Significant fibrosis (F2) Severe fibrosis (F3) Cirrhosis (F4)	241 (50.8%) 114 (24.0%) 66 (13.9%) 53 (11.2%)	65 (39.9%) 49 (30.1%) 37 (22.7%) 10 (6.1%)	0.020
Tumor size, mm	51 ± 29	30 ± 11	0.031
Number of tumors [single/multiple], n (%)	350/124 (73.8/26.2%)	127/36 (77.9/22.1%)	0.541
Tumor location [anterolateral/posterosuperior], n (%)	262/212 (55.3/44.7%)	102/61 (62.6/37.4%)	0.040
Varices	0	61 (37.4%)	0.002
Ascites	0	22 (13.5%)	0.001
Platelet count < 80 × 10^9^/L	0	67 (41.1%)	0.002
Previous liver resection, n (%)	75 (15.8%)	19 (11.6%)	0.429
Operation type Wedge resection Anatomical segmentectomy Left lateral sectionectomy Hemihepatectomy Sectionectomy and other resection	94 (19.8%) 147 (31.0%) 64 (11.4%) 132 (27.8%) 37 (7.8%)	84 (51.5%) 32 (19.6%) 12 (7.4%) 27 (16.6%) 8 (4.9%)	0.037
Iwate difficulty level Low Intermediate Advanced/Expert	152 (32.1%) 165 (34.8%) 104/53 (21.9/11.2%)	96 (58.9%) 58 (35.6%) 9/0 (5.5/0%)	0.025

**Table 2:** Conversions and reasons in MILRs for HCC in Child A and in advanced cirrhosis (Child B and Child A/B with portal hypertension) patients.

	Conv-A n = 52	Conv-B n = 21	*p* Value
Bleeding or unsatisfactory hemostasis, n (%)	21 (40.4)	11 (52.4)	0.475
Difficult adhesiolysis, n (%)	5 (9.6)	2 (9.5)	0.881
Concern of oncologic inadequacy, n (%)	20 (38.5)	6 (28.6)	0.639
Unsatisfactory Bili stasis, n (%)	4 (7.7)	1 (4.8)	0.129
Anesthesiological problems, n (%)	2 (3.8)	1 (4.8)	0.292

**Table 3:** Results of completed versus converted MILRs for HCC in Child A patients without portal hypertension.

	Compl-A n = 422	Conv-A n = 52	*p* Value
Operative time, minutes	210 (155–260)	190 (155–245)	0.488
Blood loss, mL	100 (50–160)	400 (150–570)	0.009
Red blood cell transfusion, n (%)	21 (4.9%)	3 (5.7%)	0.716
Fresh frozen plasma transfusion, n (%)	14 (3.3%)	11 (21.1%)	0.004
R0, n (%)	413 (97.8%)	50 (96.1%)	0.542
Use of Pringle maneuver, n (%)	358 (84.8%)	46 (88.5%)	0.671
Duration of Pringle maneuver, minutes	30 ± 20	40 ± 20	0.499
Total morbidity, n (%)	50 (11.8%)	12 (23.1%)	0.018
Grade 1	5 (1.2%)	2 (3.8%)	0.409
Grade 2	27 (6.4%)	7 (13.4%)	0.016
Grade 3	22 (5.2%)	3 (5.8%)	0.638
Grade 4	0	0	NC
Grade 5	0	0	NC
90-days mortality, n (%)	0	0	NC
Bleeding	8 (1.9%)	2 (3.8%)	0.841
Bile leak	17 (4.0%)	3 (5.7%)	0.778
Ascites	21 (5.0%)	9 (17.3%)	0.004
Postoperative liver failure	10 (2.4%)	5 (9.6%)	0.018
Collection	10 (2.4%)	2 (3.8%)	0.183
Chest infection	5 (1.2%)	1 (1.9%)	0.205
Pleural effusion	12 (2.8%)	4 (7.7%)	0.015
Length of stay, days	5 (3–6)	7 (5–10)	0.007
Readmissions, n (%)	8 (1.9%)	4 (7.7%)	0.036

NC: Not calculated.

**Table 4:** Results of completed versus converted MILRs for HCC in advanced cirrhosis patients (Child B and Child A/B with portal hypertension).

	Compl-B n = 142	Conv-B n = 21	*p* Value
Operative time, minutes	200 (160–280)	230 (180–290)	0.746
Blood loss, mL	250 (280–360)	550 (370–700)	0.007
Red blood cell transfusion, n (%)	5 (3.5%)	1 (4.7%)	0.655
Fresh frozen plasma transfusion, n (%)	9 (6.3%)	6 (28.6%)	0.003
R0, n (%)	138 (97.2%)	20 (95.2%)	0.903
Use of Pringle maneuver, n (%)	108 (76.0%)	17 (80.9%)	0.549
Duration of Pringle maneuver, minutes	35 ± 10	30 ± 15	0.336
Total morbidity, n (%)	18 (12.7%)	6 (28.6%)	0.002
Grade 1	6 (4.2%)	3 (14.3%)	0.032
Grade 2	10 (7.0%)	3 (14.3%)	0.021
Grade 3	2 (1.4%)	0	0199
Grade 4	0	0	NC
Grade 5	0	0	NC
90-days mortality, n (%)	0	0	NC
Bleeding	5 (3.5%)	1 (4.7%)	0.971
Bile leak	6 (4.2%)	1 (4.7%)	0.843
Ascites	7 (4.9%)	4 (19.0%)	0.008
Postoperative liver failure	4 (2.8%)	2 (9.5%)	0.034
Collection	3 (2.1%)	1 (4.8%)	0.437
Chest infection	2 (1.4%)	0	0.588
Pleural effusion	6 (4.2%)	2 (9.5%)	0.039
Length of stay, days	5 (3–7)	8 (4–10)	0.005
Readmissions, n (%)	5 (3.5%)	2 (9.5%)	0.024

NC: Not calculated.

**Table 5:** Results of converted MILRs for HCC: Child A versus converted MILRs in advanced cirrhosis (Child B and Child A/B with portal hypertension) patients.

	Conv-A n = 52	Conv-B n = 21	* p * Value
Operative time, minutes	190 (155–245)	230 (180–290)	0.574
Blood loss, mL	400 (150–570)	550 (370–700)	0.089
Red blood cell transfusion, n (%)	3 (5.7%)	1 (4.7%)	0.208
Fresh frozen plasma transfusion, n (%)	11 (21.1%)	6 (28.6%)	0.091
R0, n (%)	50 (96.1%)	20 (95.2%)	0.998
Use of Pringle maneuver, n (%)	46 (88.5%)	17 (80.9%)	0.991
Duration of Pringle maneuver, minutes	40 ± 20	30 ± 15	0.804
Total morbidity, n (%)	12 (23.1%)	6 (28.6%)	0.503
Grade 1	2 (3.8%)	3 (14.3%)	0.030
Grade 2	7 (13.4%)	3 (14.3%)	0.215
Grade 3	3 (5.8%)	0	0.622
Grade 4	0	0	NC
Grade 5	0	0	NC
90-days mortality, n (%)	0	0	NC
Bleeding	2 (3.8%)	1 (4.7%)	0.856
Bile leak	3 (5.7%)	1 (4.7%)	0.446
Ascites	9 (17.3%)	4 (19.0%)	0.101
Postoperative liver failure	5 (9.6%)	2 (9.5%)	0.923
Collection	2 (3.8%)	1 (4.8%)	0.748
Chest infection	1 (1.9%)	0	0.937
Pleural effusion	4 (7.7%)	2 (9.5%)	0.131
Length of stay, days	7 (5–10)	8 (4–10)	0.529
Readmissions, n (%)	4 (7.7%)	2 (9.5%)	0.785

NC: Not calculated.

**Table 6:** Results of converted MILRs for HCC: Child A versus converted MILRs in advanced cirrhosis (Child B and Child A/B with portal hypertension) patients for low Iwate difficulty level MILRs.

	Conv-A n = 15	Conv-B n = 8	* p * Value
Operative time, minutes	150 (130–210)	190 (150–230)	0.665
Blood loss, mL	300 (150–450)	450 (350–550)	0.183
Red blood cell transfusion, n (%)	1 (6.7%)	0	NC
Fresh frozen plasma transfusion, n (%)	2 (13.3%)	1 (12.5%)	0.912
R0, n (%)	14 (93.3%)	8 (100%)	0.832
Use of Pringle maneuver, n (%)	12 (80%)	6 (75%)	0.304
Duration of Pringle maneuver, minutes	30 ± 15	20 ± 15	0.628
Total morbidity, n (%)	3 (20%)	1 (12.5%)	0.078
Grade 1	1 (6.6%)	1 (12.5%)	0.024
Grade 2	2 (13.3%)	0	NC
Grade 3	0	0	NC
Grade 4	0	0	NC
Grade 5	0	0	NC
90-days mortality, n (%)	0	0	NC
Bleeding	1 (6.6%)	0	NC
Bile leak	0	1 (4.7%)	NC
Ascites	1 (6.6%)	0	NC
Postoperative liver failure	0	0	NC
Collection	1 (6.6%)	0	NC
Chest infection	0	0	NC
Pleural effusion	0	0	NC
Length of stay, days	6 (5–11)	7 (5–11)	0.779
Readmissions, n (%)	1 (6.6%)	1 (4.7%)	0.625

**Table 7:** Results of converted MILRs for HCC: Child A versus converted MILRs in advanced cirrhosis (Child B and Child A/B with portal hypertension) patients for intermediate/expert/advanced Iwate difficulty level MILRs.

	** Conv-A ** ** n = 37 **	** Conv-B ** ** n = 13 **	*** p *** ** Value **
Operative time, minutes	210 (170–280)	250 (190–300)	0.227
Blood loss, mL	400 (270–520)	700 (400–900)	0.029
Red blood cell transfusion, n (%)	2 (5.4%)	1 (7.7%)	0.034
Fresh frozen plasma transfusion, n (%)	9 (24.3%)	5 (38.5%)	0.007
R0, n (%)	36 (97.3%)	12 (92.3%)	0.905
Use of Pringle maneuver, n (%)	34 (91.9%)	11 (84.6%)	0.076
Duration of Pringle maneuver, minutes	45 ± 25	40 ± 10	0.765
Total morbidity, n (%)	9 (24.3%)	5 (38.5%)	0.038
Grade 1	1 (2.7%)	2 (15.4%)	0.020
Grade 2	5 (13.5%)	3 (23.1%)	0.031
Grade 3	3 (8.1%)	0	NC
Grade 4	0	0	NC
Grade 5	0	0	NC
90-days mortality, n (%)	0	0	NC
Bleeding	0	1 (7.7%)	NC
Bile leak	3 (8.1%)	0	NC
Ascites	8 (21.6%)	4 (30.8%)	0.025
Postoperative liver failure	5 (13.5%)	2 (15.4%)	0.535
Collection	1 (2.7%)	1 (7.7%)	0.034
Chest infection	1 (2.7%)	0	NC
Pleural effusion	4 (10.8%)	2 (15.4%)	0.037
Length of stay, days	6 (5–10)	9 (4–10)	0.022
Readmissions, n (%)	3 (8.1%)	1 (7.7%)	0.809

## Data Availability

The data presented in this study are available on request from the corresponding author. The data are not publicly available due to privacy restrictions.
